# Can otoplasty impact hearing? A prospective randomized controlled study examining the effects of pinna position on speech reception and intelligibility

**DOI:** 10.1186/1916-0216-42-10

**Published:** 2013-02-02

**Authors:** Michael L McNeil, Steve J Aiken, Manohar Bance, Jeff R Leadbetter, Paul Hong

**Affiliations:** 1 Division of Otolaryngology-Head and Neck Surgery, Department of Surgery, Dalhousie University, Halifax, Nova Scotia, Canada; 2 School of Human Communication Disorders, Faculty of Health Sciences, Dalhousie University, Halifax, Nova Scotia, Canada; 3 SENSE Lab, QEII Health Sciences Centre, Halifax, Nova Scotia, Canada; 4 IWK Health Centre, Halifax, Nova Scotia, Canada

## Abstract

**Objectives:**

Otoplasty is a commonly performed surgical procedure that restores the ideal position of the pinna. Although the pinna is a well-recognized component of the auditory apparatus, no studies have assessed the audiological effects of this procedure. We sought to quantify the impact of pinna repositioning on speech intelligibility and reception.

**Methods:**

Eighteen adults with normal hearing and pinnae were recruited and the pinna positions were randomized in each participant. Intracanal acoustical analysis was performed to calculate the Speech Intelligibility Index (SII). Hearing In Noise Test (HINT) with two azimuth speaker arrangement was also performed. The outcome measures were compared using paired *t*-tests for both pinna positions.

**Results:**

The SII significantly improved with the pinna in forward position (49.3 vs. 45.8, p<0.001). HINT thresholds also improved with the pinna forward (-6.43dB vs. -5.08dB, p=0.0003).

**Conclusions:**

Pinna position affects audiological performance, in both speech intelligibility and speech reception in noise. These are novel findings that may impact the informed consent process and decision to treat for patients undergoing otoplasty.

## Background

Protruding ears, or prominotia, is the most common congenital deformity of the head and neck, with an incidence as high as 5 percent in certain populations [1]. The specific etiology of the protruding ears is usually due to an underdeveloped antihelical fold and/or conchal bowl hypertrophy [2]. Otoplasty is a surgical procedure, employed primarily in children, that attempts to restore the ideal aesthetic position of the pinna.

Many studies have described the “ideal” position of the outer ear [1-4]. In addition, there are several anthropometric studies that document normal auricular dimensions throughout childhood [5-7].

Protrusion of the pinna is typically measured along a hypothetical plane drawn from the lateral helical margin to the mastoid scalp. From the scalp, the helical rim at the superior pole normally projects laterally 10 to 12 mm, at the midpoint 16 to 18 mm, and at the lobule 20 to 22 mm [8,9]. The auriculocephalic angle, which is normally less than 35 degrees, has also been used to describe the degree of auricular protrusion [8,10].

The pinna is a well-recognized component of the auditory system, with the ability to shape the incoming acoustic signal. Several studies have demonstrated that the shape of the pinna affects spatial localization, mainly in the vertical plane [11-13]. In everyday life, however, the ability to understand speech in background noise impacts functional hearing more than the locating zenith for sound. Despite this, no studies have assessed the effects of pinna position on speech intelligibility and perception, despite some evidence that the shape and position of the external ear may affect these audiometric measures [14].

The literature on otoplasty has concentrated on technical considerations with numerous articles focusing on various surgical methods to correct the outstanding ear [3,4,15]. Interestingly, no studies were identified that have assessed any aspects of hearing in relation to otoplasty procedures.

In this study, we investigated the effects of pinna position changes on speech intelligibility and reception in normal hearing adult subjects. We used the Hearing in Noise Test (HINT) materials to assess speech comprehension. To understand the acoustic effects, we also examined the pinna to external ear canal transfer function, and how it is changed by pinna position, and what the impact of this would be on the calculated speech intelligibility index (SII), and to see if this agreed with the measured HINT for different pinna positions.

There are two ways that changing pinna position may affect speech comprehension in background noise. When speech and noise are presented together in space, the signal to noise (S/N) level required to understand speech is higher than if the noise and speech are separated, this is known as spatial release from masking [16]. This is a binaural process, requiring central processing. However, the pinna also increases sound pressure near the ear canal for sound incident from the front (baffle effect), and attenuates it for sound coming from behind (shielding effect). Depending on where speech and noise are relative to the pinna, changing pinna position may affect the ratio of the S/N amplitude. This will also increase or decrease speech comprehension in noise, but is purely a monaural effect requiring no central binaural computing. In this study, we altered both by changing the pinna position.

## Methods

The study protocol was reviewed and approved by the local Institutional Review Board.

Seventeen adults with normal hearing and normal pinna shape and position were recruited. The participants were medical students and residents enrolled at Dalhousie University (Halifax, Nova Scotia) or ear research laboratory employees. All potential candidates were given a brief explanation of the study, screened for a history of hearing loss, then invited to participate. If agreeable, they were given a study information sheet and asked to sign an informed consent form. Participants were not offered remuneration for their involvement.

**Figure 1:**
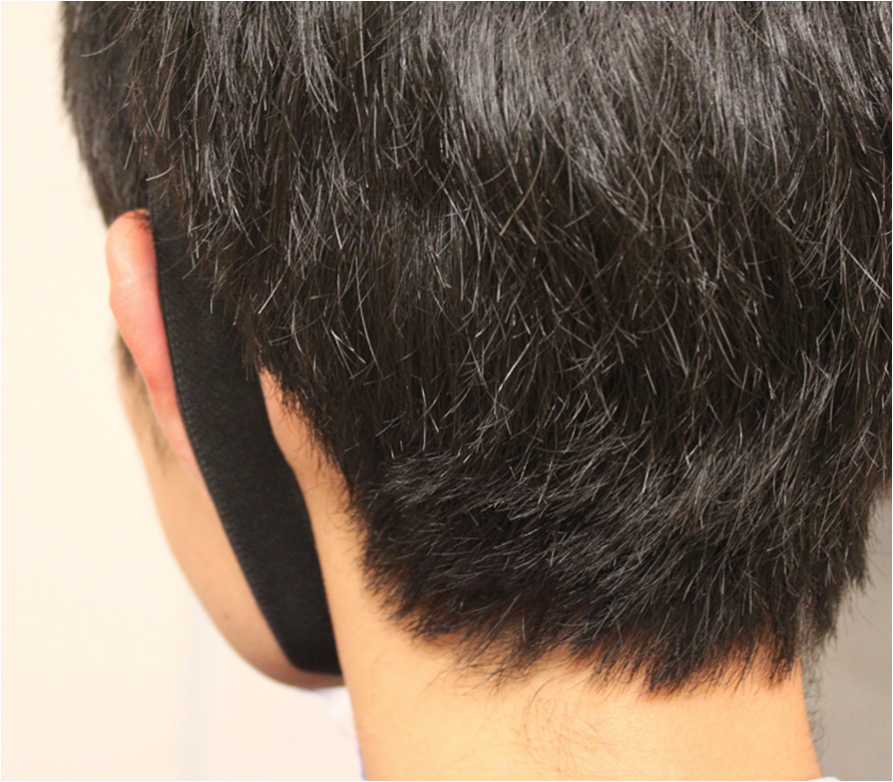
Subject with left pinna projected forward using Baha^®^ soft band.

For each subject, tests were performed four times: twice each with the pinna in “neutral” or their native position and “forward” or protruded position. To simulate a protruding pinna position, a bone-anchored hearing aid (Baha^®^) soft band was placed around the head, behind the left auricle (Figure 1). More specifically, the Baha^®^ soft band was placed posteriorly just medial to the scapha to protrude both the helical rim and the conchal bowl. The position of the pinna (in neutral and forward positions) was measured from the lateral aspect of the helical rim to the mastoid skin at the mid-level (half way between the superior pole and the cauda helix) of the outer ear using a surgical caliper.

### Audiometric Testing

Screening audiometry was performed on each subject to ensure normal hearing. A standard 25 dB pure tone was introduced into each ear at 500, 1000, 2000, 4000, and 8000 Hz. Positive identification of all tones in both ears constituted a successful screening test. All recruited subjects were deemed to have normal hearing.

The remainder of the tests were performed solely with the left ear to shorten the overall testing time and subject compliance. Given that in all subjects, both ears had normal screening audiometry results, it was likely that similar results would be found regardless of the ear tested.

### Speech Intelligibility Index (SII)

An Audioscan Verifit VF system (Etymonic Design Inc., Dorchester, Ontario, Canada) was used to measure the sound pressure in the ear canal in both pinna positions, and to calculate the SII in accordance with ANSI S3.5, 1997, R2007. The SII is the predicted speech intelligibility, based on audibility of the speech signal at different frequencies. The SII was calculated by the Verifit software on the basis of the sound pressure level induced by a 12.8 second speech signal (Verifit Test Signal #1) measured within 5 mm of the tympanic membrane, with the left pinna in both neutral and forward position. The order was randomized using Microsoft Excel for Mac 2011 (Redmond, Washington, USA) by using the rand() function.

In a soundproof booth, the Audioscan Verifit VF was positioned five feet from a wall to avoid reflections. The sensor was appropriately calibrated using the standard Verifit procedure. The volunteer was positioned in a chair three feet directly in front of the Verifit, and an insert microphone was placed in their left ear, once each with the pinna in neutral and forward positions. The placement of the distal probe tip was confirmed with otoscopy.

As eluded to above, the SII provides an estimate of predicted speech intelligibility on the basis of the level of speech above background noise, and hearing thresholds (i.e. speech audibility) in a number of importance-weighted frequency bands (ANSI S3.5-1997). Because the measurements were made with normally hearing subjects in a sound-attenuating booth, speech levels were much higher than hearing thresholds and background noise levels were negligible. As a consequence, small differences in speech power levels resulting from changes in pinna position would not change the SII estimate due to ceiling effects. However, real-world listening rarely takes place in such quiet environments and many individuals have some degree of hearing loss, so small changes in speech audibility resulting from pinna position differences could affect speech audibility, and hence intelligibility. In order to improve the sensitivity of the SII to small changes in level, the subjects’ hearing threshold was set to 40 dB HL at all audiometric frequencies for the SII calculation. This means, the audibility and predicted speech comprehension was calculated for a person whose hearing threshold was assumed to be 40 dB HL.

The stimulus level was set to 65 dB SPL(A) and the ear canal sound pressure for the 12.8 second stimulus was measured in each subject four times; twice with the pinna in each position.

### Hearing in Noise Test (HINT)

The HINT is a standardized test for assessing speech perception in noise, and can be used for measuring the improvement in speech perception that occurs when noise and speech come from different directions instead of the same direction (i.e., the ‘spatial release from masking’) [17]. During the test, listeners are instructed to repeat sentences that are presented from a speaker at 0° azimuth, while noise is presented at azimuths of 0°, +90° (right), and -90° (left), in different conditions. As per the standard HINT protocol, the noise is held constant at a level of 65 dBA, while the speech is varied to find the threshold of 50% intelligibility. HINT thresholds are expressed as speech-to-noise ratios (in dB). The release from masking (although in this case actually the sum of central release from masking, and changes due to changes in S/N levels in the left ear) is usually measured by the difference between the HINT thresholds with noise at 0° (i.e. speech and noise spatially coincident) and with noise at plus or minus 90° azimuths (i.e. with speech and noise spatially separated). When listening binaurally (i.e., with two ears), normally hearing listeners can use the binaural information to separate the speech from the noise signal, and show an average release from masking of about 7 dB when noise and speech are separated [18].

**Figure 2:**
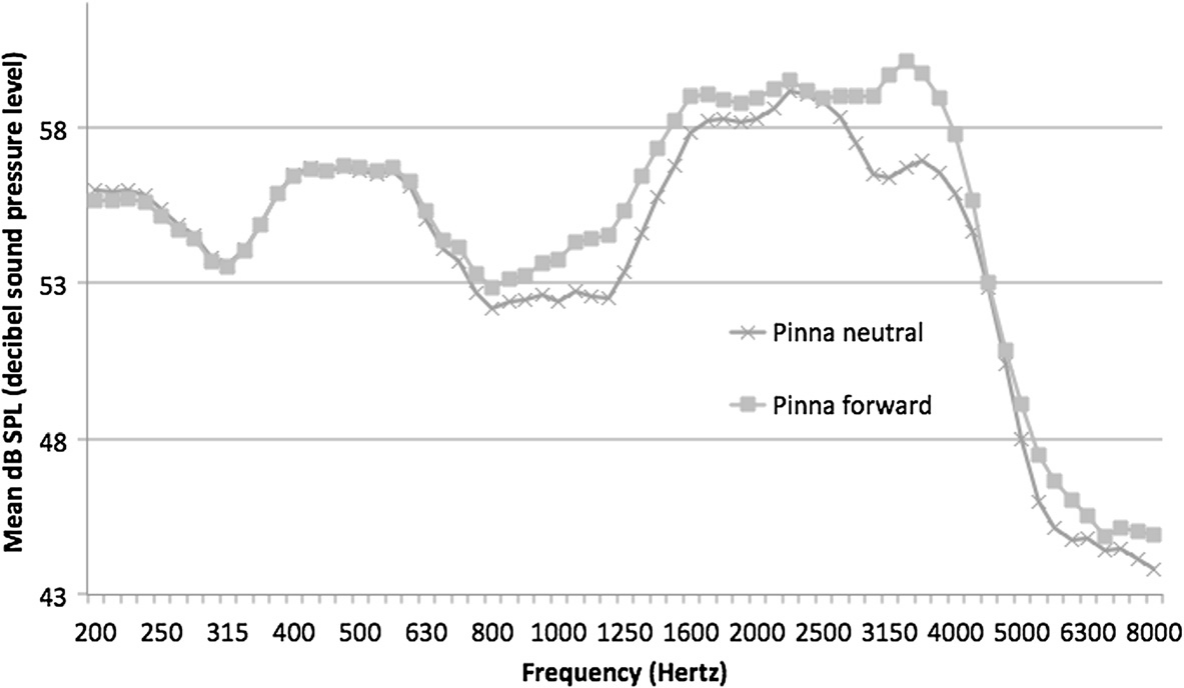
Speech spectrum measured at tympanic membrane for pinna neutral and forward positions.

In the present study, the HINT test was performed on each subject with the left pinna in neutral and forward positions, with the speech presented at 45° to the left, and the noise presented at left 45° (coincident with speech) and left 135° (90° separated from speech) in different conditions. The right pinna was unaltered and not occluded. Phillips and colleagues [19] found a small release from masking (1.27 dB) under these conditions with the pinna in neutral position (i.e., with speech presented at 45°, the HINT threshold was 1.27 dB lower when noise was presented at 135° than when presented at 45°). Moving the pinna to a forward position might be expected to increase the acoustic intensity in the ear canal for sound emanating from 45° (baffle effect), while decreasing the acoustic energy in the ear canal for sounds presented at 135° (shield effect). This should result in increased S/N ratio at the tympanic membrane with speech at 45° and noise at 135°. Average speech spectrum acoustic levels at the tympanic membrane are shown in Figure 2 as a function of pinna position.

**Figure 3:**
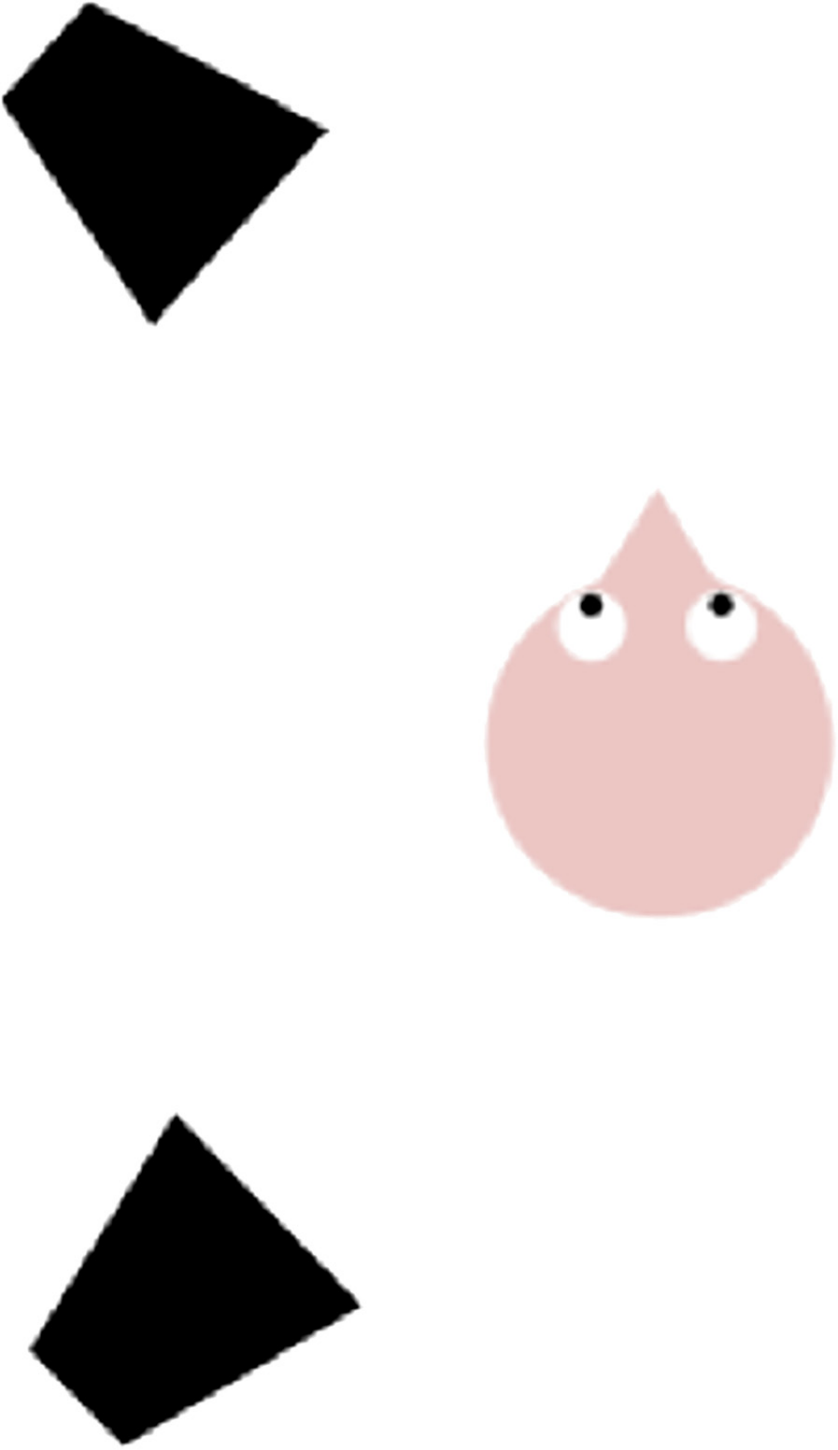
**Speaker arrangement for Hearing in Noise Test (HINT).** The left anterior speaker (45 degrees from direct anterior) was used for speech delivery in all tests, and noise delivery in half of the test cases. The left posterior speaker (135 degrees) was used for noise delivery in the other half of test cases.

The sound booth was arranged with speakers located at 45° and 135° left from the patient’s midline (Figure 3). The right ear was not occluded, and it’s pinna position was not altered for any test conditions. As the speech and noise sources were introduced on the subject’s left side, the contribution of the right pinna would be minimal. Furthermore, as a prospective study, we chose to focus on a small number of conditions where the acoustic effects would likely be largest, and therefore, monaural testing was conducted.

HINT testing was performed using a GSI 61 Clinical Audiometer calibrated in accordance with ANSI S3.6-2004.

Twenty-five standard HINT sentence lists were used, each comprising 20 sentences. Four randomized lists were presented to each subject. The sentences were projected from the speaker at -45° to the subject (i.e. eccentrically forward to the subjects test ear).

Speech tests were performed in both pinna positions, and with background noise either coming from the same speaker (i.e. speech and noise both at -45° to the subject), and with speech at -45° and noise at -135° (i.e. noise from in front or behind the variably cupped pinna). As mentioned above, the pinna acted as a shield to the noise in the -135° condition, and a baffle in the -45° condition. The four subtests performed were thus, HINT score with speech at -45° and:

- Test A: Pinna neutral, noise at -45°

- Test B: Pinna neutral, noise at -135°

- Test C: Pinna forward, noise at -45°

- Test D: Pinna forward, noise at -135°

The order of the subtests was randomized for each subject. The subjects were not pre-informed of the direction of the noise before each randomized subtest. Blinding the subject to their pinna position was not possible due to the tactile nature of the softband.

During each subtest, the noise was introduced at 65 dB SPL(A) and kept constant at this level. The first sentence list was introduced at 65 dB SPL(A) as well, while the subsequent three sentences were introduced at 5 dB louder if the subject failed to successfully repeat the initial list, or less by 5 dB if they were successful. The remaining 16 sentences were increased or decreased by 2 dB depending on the ability to correctly repeat sentences. The S/N level at which 50% correct responses were obtained was recorded.

The mean sentence level setting *after* tests 4 through 20 was calculated, as per the standard HINT scoring protocol. The HINT threshold (dB S/N) for each subtest was calculated as mean sentence level (dB) minus 65 dB SPL(A). It is important to appreciate that even small changes in the S/N ratio in this test can translate to large changes in sentence recognition scores (e.g., see Figure 3 in Sherbecoe & Studebaker, 2002 [20]).

### Statistical Analysis

Paired, two-tailed *t*-tests were used to compare the helical rim-to-mastoid distance, HINT, and SII results for the neutral and forward pinna positions. Pearson correlations were calculated to compare the relationship among these data.

SII was also compared to HINT data using paired, two-tailed *t*-tests. The relationship between the difference in HINT scores with pinna in two positions, and the difference in predicted change in HINT score (by calculated SII) by pinna position was examined by *t*-tests.

## Results

### Subject Demographics

Seventeen volunteers (ten males, seven females; ages 22 to 43 years) were recruited. Their baseline demographics are outlined in Table 1. None of the subjects had undergone any external, middle, or inner ear surgeries in the past. The mean helical rim-to-mastoid distances were: neutral pinna position, 17.8 mm (range 11 to 22 mm), and pinna forward position 23.3 mm (range 18 to 27 mm). In all subjects, measured pinna distances increased with pinna being artificially protruded with the Baha^®^ softband, as expected (mean change = 5.5 mm, range 1 to 8 mm). The increase was statistically significant (*p* = 0.0000).

**Table 1:** Patient characteristics and pinna measurements

**Subject**	**Age**	**M/F**	**Helix-to-mastoid distance (mm)**		
			**Neutral**	**Forward**	**Difference**
1	34	M	21	22	1
2	33	M	22	26	4
3	28	M	19	25	6
4	27	M	20	27	7
5	26	M	22	26	4
6	26	M	20	23	3
7	37	F	11	19	8
8	28	F	19	23	4
9	42	F	15	19	4
10	35	F	11	18	7
11	36	M	20	26	6
12	43	F	15	22	7
13	40	M	17	25	8
14	25	F	18	25	7
15	33	F	17	23	6
16	22	M	15	21	6
17	28	M	21	26	5
**Mean**	**31.9**		**17.8**	**23.3**	**5.5**

M: male, F: female, mm: millimeters, Neutral: pinna in neutral position, Forward: pinna in forward position.

### Speech Intelligibility Index

**Figure 4:**
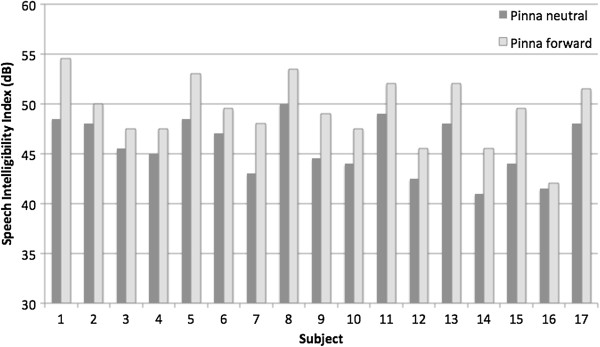
Calculated speech intelligibility index (SII) for each subject with pinna in neutral and forward positions.

The calculated SII significantly improved with the pinna in forward position (49.3 versus 45.8, *p* < 0.001). The SII data are summarized in Table 2, and demonstrated in Figure 4.

**Table 2:** Speech intelligibility index with pinna in neutral and forward positions

**Subject**	**Neutral**	**Forward**	**Difference**
1	48.5	54.5	*6.0*
2	48.0	50.0	*2.0*
3	45.5	47.5	*2.0*
4	45.0	47.5	*2.5*
5	48.5	53.0	*4.5*
6	47.0	49.5	*2.5*
7	43.0	48.0	*5.0*
8	50.0	53.5	*3.5*
9	44.5	49.0	*4.5*
10	44.0	47.5	*3.5*
11	49.0	52.0	*3.0*
12	42.5	45.5	*3.0*
13	48.0	52.0	*4.0*
14	41.0	45.5	*4.5*
15	44.0	49.5	*5.5*
16	41.5	42.0	*0.5*
17	48.0	51.5	*3.5*
**Mean**	**45.8**	**49.3**	**3.5**

Neutral: pinna in neutral position, Forward: pinna in forward position.

**Figure 5:**
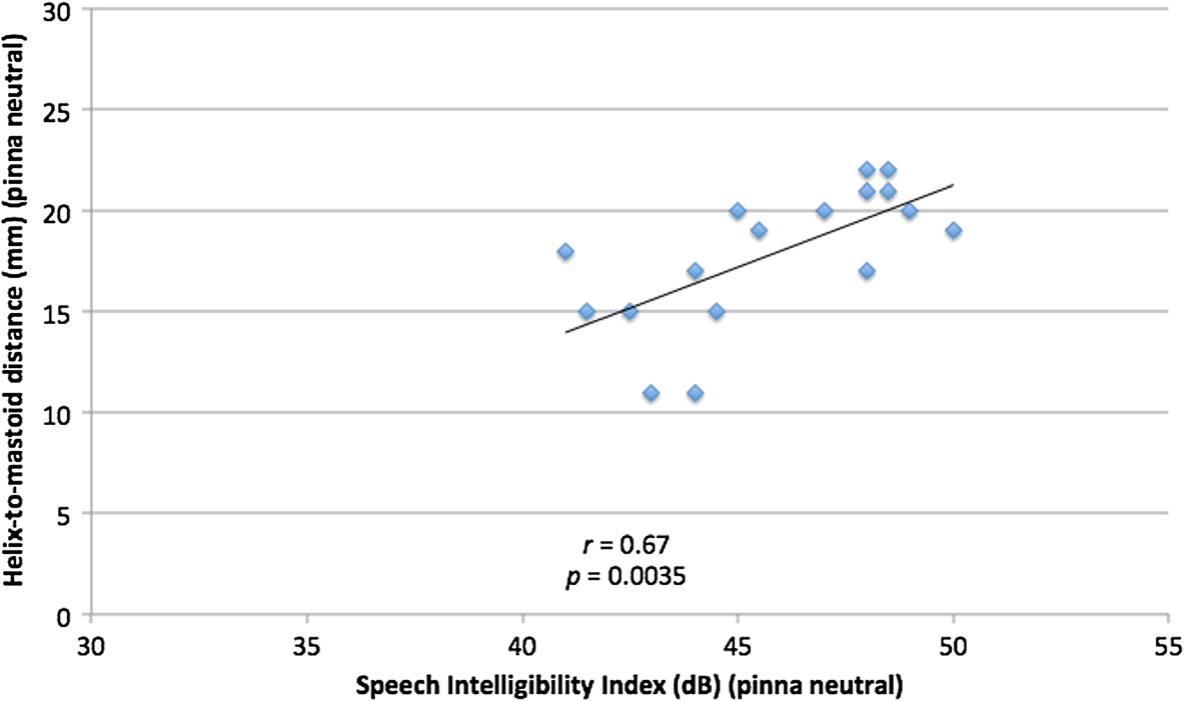
Comparison of helix-to-mastoid distance (mm) and Speech Intelligibility Index (dB) for pinna in neutral position.

**Figure 6:**
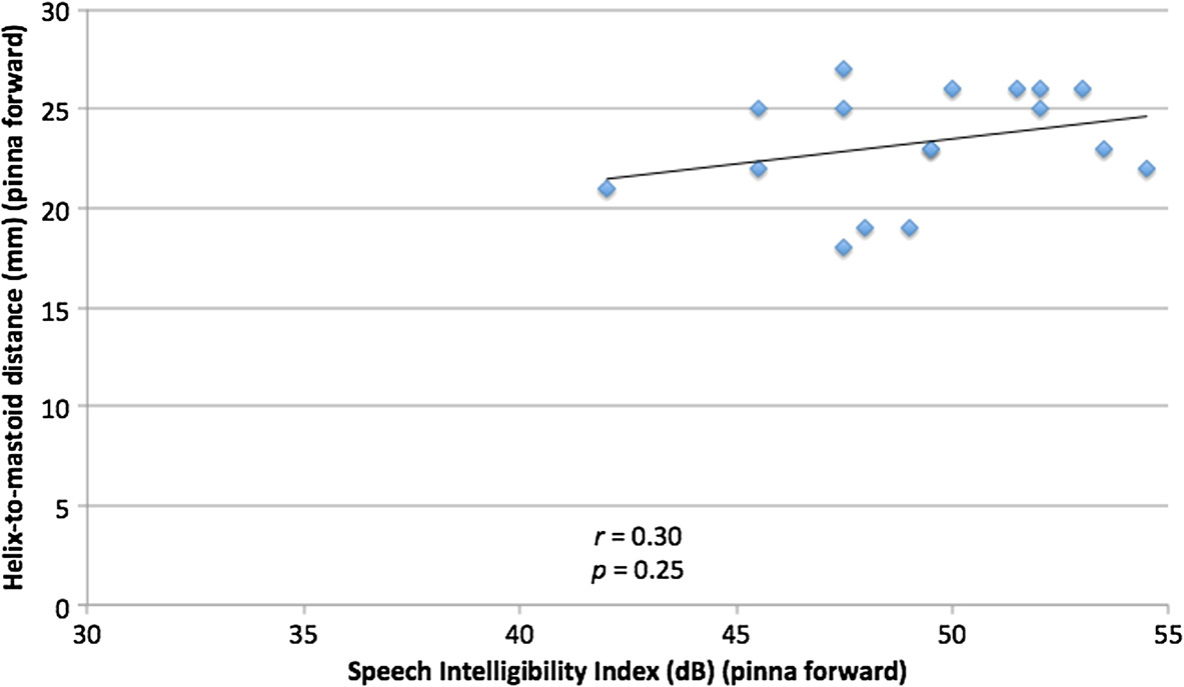
Comparison of helix-to-mastoid distance (mm) and Speech Intelligibility Index (dB) for pinna in forward position.

To examine the relationship of absolute ear position and SII across all subjects, Pearson correlation coefficient between predicted SII and neutral pinna-to-mastoid distance was calculated, which was found to be 0.67, reflecting a moderate correlation (*p* = 0.0035) (Figure 5). When examining the correlation between predicted SII and pinna-to-mastoid distance with pinna forward, the correlation was no longer statistically significant (*r* = 0.30, *p* = 0.25) (Figure 6).

### Hearing in Noise Test

With noise at -135°, the measured HINT S/N ratio with the pinna forward was 1.35 dB better compared to when the pinna was in the neutral position (-6.43 dB versus -5.08 dB, *p* = 0.0003). Similarly, with noise at -45°, pinna forward position had a lower (better) 0.77 dB S/N ratio (2.87 dB versus 2.10 dB*, p* = 0.0092). These data are summarized in Table 3.

**Table 3:** Hearing in noise test outcomes under different conditions

**Subject**	**HINT mean dB(A) SPL**					
	**Neutral, Noise 45°**	**Neutral, Noise 135°**	**Forward, Noise 45°**	**Forward, Noise, 135°**	**45° Δ**	**135° Δ**
1	59.59	57.35	58.41	55.82	*1.18*	*1.53*
2	58.65	55.59	56.53	53.00	*2.12*	*2.59*
3	57.94	54.41	57.00	54.18	*0.94*	*0.24*
4	57.59	59.94	57.71	58.18	*−0.12*	*1.76*
5	56.53	55.12	57.00	53.47	*−0.47*	*1.65*
6	57.47	53.24	56.06	52.29	*1.41*	*0.94*
7	58.29	53.24	56.29	52.53	*2.00*	*0.71*
8	58.76	56.76	57.00	57.24	*1.76*	*−0.47*
9	59.29	55.10	58.71	54.14	*0.57*	*0.95*
10	57.95	54.14	56.62	52.71	*1.33*	*1.43*
11	58.05	54.81	56.52	53.19	*1.52*	*1.62*
12	57.67	54.33	59.86	55.86	*−2.19*	*−1.52*
13	56.24	54.05	55.95	51.67	*0.29*	*2.38*
14	58.41	53.71	57.24	50.76	*1.18*	*2.94*
15	57.94	53.71	56.53	52.76	*1.41*	*0.94*
16	57.24	53.47	57.00	51.82	*0.24*	*1.65*
17	56.76	54.65	56.76	51.12	*0.00*	*3.53*
**Mean**	**57.90**	**54.92**	**57.13**	**53.57**	**0.77**	**1.34**

HINT: Hearing in Noise Test, dB(A): A-weighted decibel, SPL: sound pressure level, Neutral: pinna in neutral position, Forward: pinna in forward position, Noise 45°: noise presented at 45 degrees left from center forward, Noise 135°: noise presented at 135 degrees left from center forward, Δ: change in HINT between pinna neutral and pinna forward.

**Figure 7:**
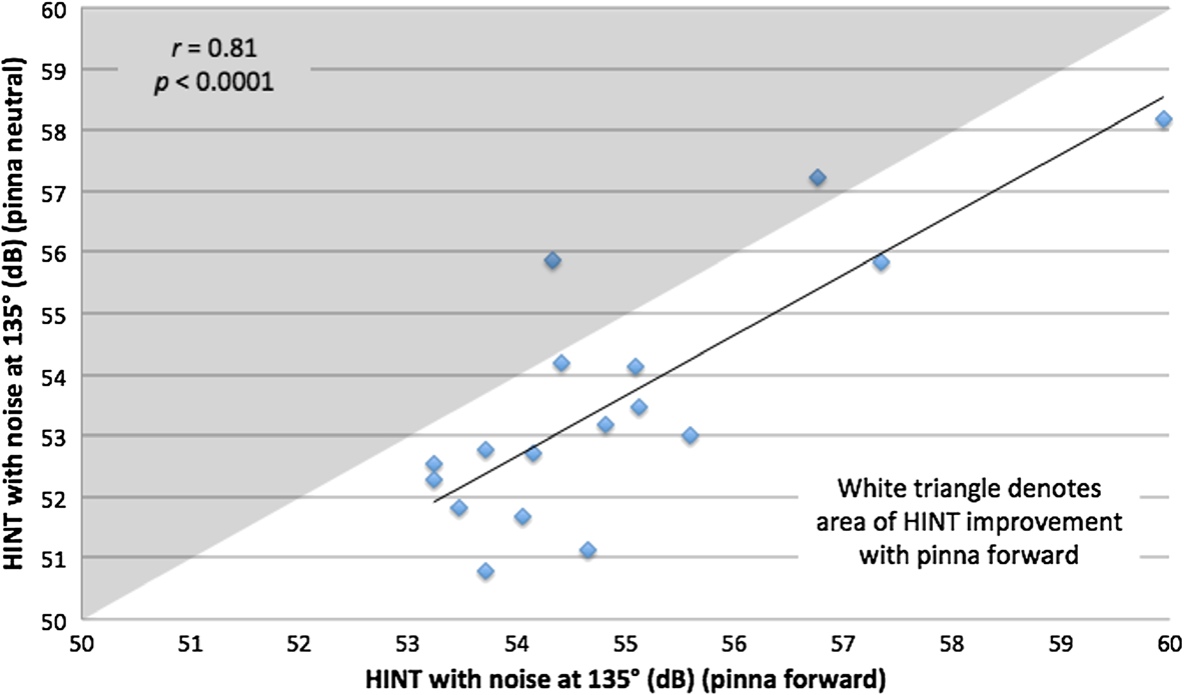
**Hearing in noise test (HINT) with noise at 135 degrees, comparing pinna in neutral and in forward positions.** Diamonds in white triangle denotes HINT scores in two conditions, and generally show improvement in HINT score with pinna forward.

**Figure 8:**
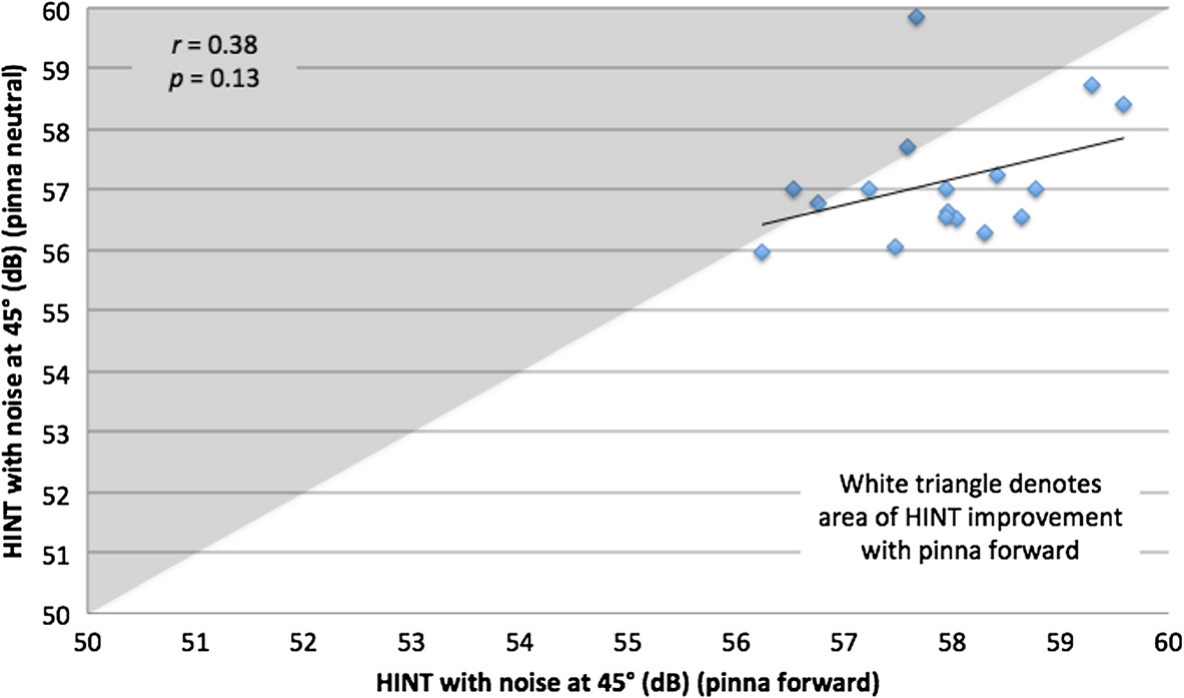
**Hearing in noise test (HINT) with noise at 45 degrees and pinna in neutral and in forward positions.** Diamonds in white triangle denotes improvement in HINT score with pinna forward.

The Pearson correlation between HINT with pinna forward and neutral positions for individual subjects was strong (*r* = 0.81, *p* < 0 .0001) with noise at -135°, so that subjects showed good intra-subject correlation in the two conditions. This relationship is demonstrated in Figure 7. The correlation with noise at -45° (Figure 8) was moderate (*r* = 0.38) but not statistically significant (*p* = 0.13).

### Comparison of SII and HINT

**Figure 9:**
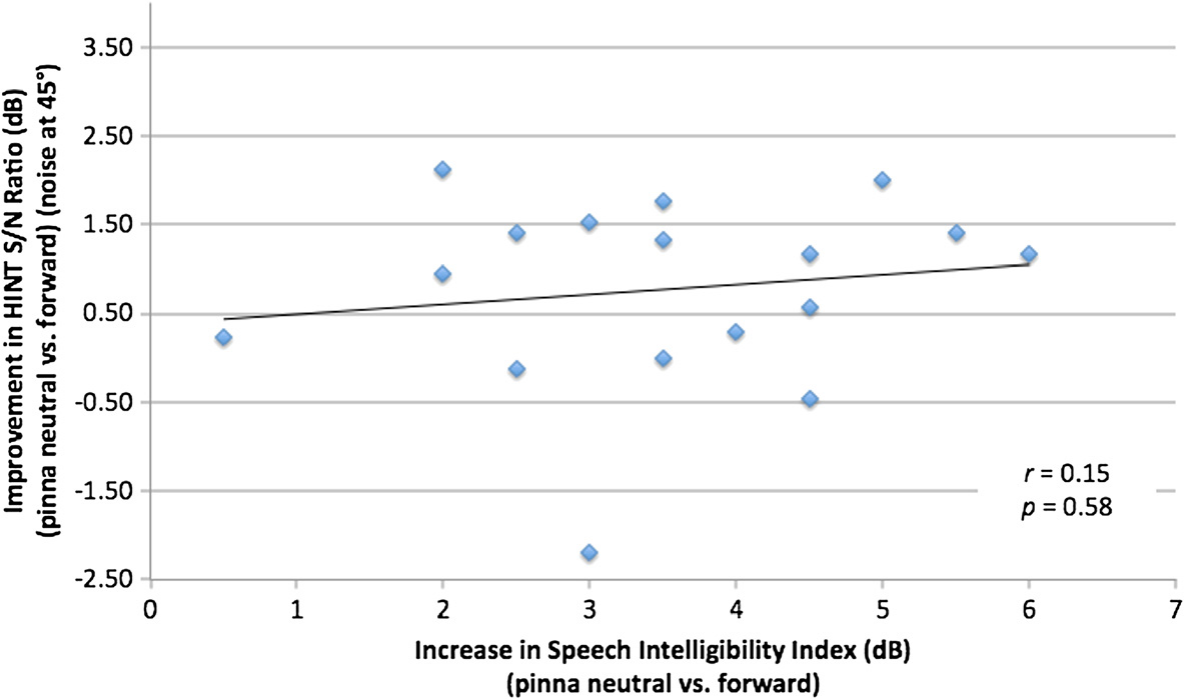
Comparison of change in hearing in noise test (HINT) signal-to-noise (S/N) ratio (pinna neutral vs. forward) with noise presented at 45 degrees vs. change in speech intelligibility index (pinna neutral vs. forward).

**Figure 10:**
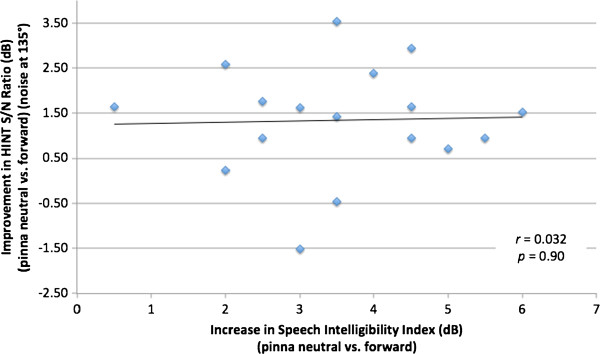
Comparison of change in hearing in noise test (HINT) signal-to-noise (S/N) ratio (pinna neutral vs. forward) with noise presented at 135 degrees vs. change in speech intelligibility index (pinna neutral vs. forward).

The release from masking (by change in pinna position between HINT tests) was compared with the change in SII between pinna positions. When the HINT noise was introduced at -45°, there was not a significant correlation (*r* = 0.15, *p* = 0.58) (Figure 9). Similarly, the comparison of changes in HINT with noise at -135° and change in SII was not statistically significant (*r* = 0.032, *p* = 0.90) (Figure 10).

## Discussion

The treatment of prominent ears with otoplasty is a relatively common procedure. The International Society of Aesthetic Plastic Surgery estimated that over 242,000 otoplasties were performed worldwide in 2010 [21]. At high volume institutions, otoplasty results in low rates of complications, and high cosmetic satisfaction [22].

By the age of 5 years, the growth of the cartilaginous pinna is almost complete [23]. This corresponds to the age when many children begin school-based education, and may be subject to stigma and ridicule by their peers. While protruding ears may be considered a sign of good fortune by some Asians, in most other cultures, they are associated with feelings of anxiety, social discomfort, and even abnormal behavior [24].

While Down syndrome and Turner syndrome may be associated with both prominent ears and conductive hearing loss, prominent ears are not generally thought to be associated with hearing issues. As well, many surgeons who perform otoplasties are unaware of any hearing related consequences with respect to the protruding ears. Thus, prominent ears in a child is not in itself an indication for any type of audiometric evaluation. This has led to the absence of any studies being conducted in this particular area.

In this surrogate study, we investigated the effects of temporary changes in the forward position of the pinna on speech intelligibility and reception in normal hearing adult subjects. While otoplasty is more commonly performed in children, adults are more cooperative and reliable subjects; they are better to accommodate a long battery of audiologic tests. While it is unlikely that the purely acoustic effects of pinna position vary from adults to children, further testing in a pediatric population is necessary to confirm the present results.

Although the function of hearing has traditionally been considered unimportant and unaltered in otoplasty procedures, this has simply been an assumption. As hearing and speech development occurs early in life and since otoplasty is performed mainly in young children, it may be important to understand how, if at all, this procedure may affect auditory function.

If “normalizing” the shape of the ear is found to affect auditory perception, this may have consequences to the child functioning in noisy environments, and maybe something further to consider in the informed consent, and risk/benefit assessment.

The shape of the pinna is thought to contribute to vertical sound localization in humans by providing monaural spectral cues. Hofman and Opstal [13] used pinna-shaping molds to demonstrate that localization accuracy is degraded with changes to the pinna, but this skill was reacquired over time. As vertical localization has limited utility in humans, we chose not to investigate this phenomenon as part of our study. Also, while the brain can learn to reinterpret changes in the acoustic frequency shaping that altered pinna may cause, and relearn localization cues, changing the pinna shape will reduce or improve the S/N ratio, which cannot be compensated for by central mechanisms. It is true, however, that portion of release from masking that occurs because of central binaural processing might be reacquired through relearning.

Our findings demonstrate a statistically significant difference in speech intelligibility. However, it is difficult to precisely quantify the degree of noticeable change in a real world scenario. It is likely to be small in the practical sense. Furthermore (although not tested in our study), there may be an adaptability to the changes in hearing after otoplasty surgery, which may affect long-term outcomes. Potential adaptation might occur in a manner similar to Hofman and Opstal’s vertical sound localization study [13] previously discussed.

Again, we found that a pinna in forward position results in a statistically significant improvement in speech intelligibility when the speech is introduced at -45° from anterior. Further investigation may find that a forward pinna may be disadvantageous for understanding speech introduced from behind. Yet, in most real world scenarios, the sound source of interest is in the front hemifield (e.g. facing your conversation partner). We chose to focus on this more likely test condition, but it is important to acknowledge that there may be scenarios in which improved rear speech intelligibility may be beneficial with the pinna in non-protruded position (e.g., hearing people in the backseat while driving a car).

This is the first study to assess any aspect of auditory function with respect to changes in pinna position. Future research may investigate children undergoing the actual otoplasty procedure, to assess both the immediate auditory effect and the long-term hearing function and possible adaption.

### Limitations

During conduction of the SII and HINT tests, neither the tester nor subjects were blinded to pinna position.

For cases of otoplasty where a conchomastoid suture is placed, there may be slight narrowing of the external auditory canal meatus. This possible effect was not reproduced in the study population and therefore, may confer the results ungeneralizable to those patients who receive the conchomastoid suture. Yet the incidence of significant meatal stenosis after otoplasty is extremely rare and therefore, this particular effect is deemed to be negligible.

## Summary

Pinna position changes affected audiological performance, in both speech intelligibility and speech reception in noise, in our study population. These are novel findings that should warrant further research and may possibly impact the informed consent process for patients undergoing otoplasty.

Accepted for publication for the Journal of Otolaryngology-Head and Neck Surgery, October 2012.Accepted for presentation at the 2012 Poliquin Resident Research Competition, Canadian Society of Otolaryngology – Head and Neck Surgery’s 66th Annual Meeting, Toronto, Ontario, Canada, May 2012.

## Competing interests

The authors declare that they have no competing interests.

## Authors’ contributions

PH, SJA, MLM and MB were involved in the study design, data analysis and interpretation. MLM and JRL carried out the experiments. All authors read and approved the final manuscript.
